# Additive Fabrication and Characterization of Biomimetic Composite Bone Scaffolds with High Hydroxyapatite Content

**DOI:** 10.3390/gels7030100

**Published:** 2021-07-23

**Authors:** Hoyeol Lee, Jin Myoung Yoo, Seung Yun Nam

**Affiliations:** 1 Industry 4.0 Convergence Bionics Engineering, Pukyong National University, Busan 48513, Korea; hoyeollee11@gmail.com (H.L.); jinmyoungyoo@gmail.com (J.M.Y.); 2 Department of Biomedical Engineering, Pukyong National University, Busan 48513, Korea

**Keywords:** 3D bioprinting, composites, hydroxyapatite, bone regeneration

## Abstract

With the increased incidence of bone defects following trauma or diseases in recent years, three-dimensional porous scaffolds fabricated using bioprinting technologies have been widely explored as effective alternatives to conventional bone grafts, which provide cell-friendly microenvironments promoting bone repair and regeneration. However, the limited use of biomaterials poses a significant challenge to the robust and accurate fabrication of bioprinted bone scaffolds that enable effective regeneration of the target tissues. Although bioceramic/polymer composites can provide tunable biomimetic conditions, their effects on the bioprinting process are unclear. Thus, in this study, we fabricated hydroxyapatite (HA)/gelatin composite scaffolds containing large weight fractions of HA using extrusion-based bioprinting, with the aim to provide an adequate biomimetic environment for bone tissue regeneration with compositional and mechanical similarity to the natural bone matrix. The overall features of the bioprinted HA/gelatin composite scaffolds, including rheological, morphological, physicochemical, mechanical, and biological properties, were quantitatively assessed to determine the optimal conditions for both fabrication and therapeutic efficiency. The present results show that the bioprinted bioceramic/hydrogel scaffolds possess excellent shape fidelity; mechanical strength comparable to that of native bone; and enhanced bioactivity in terms of cell proliferation, attachment, and osteogenic differentiation. This study provides a suitable alternative direction for the fabrication of bioceramic/hydrogel-based scaffolds for bone repair based on bioprinting.

## 1. Introduction

The aging global population has resulted in an increased incidence of bone defects following trauma or diseases in recent years [1,2,3]. For instance, the most common type of bone disease, osteoporosis, is estimated to affect more than 200 million people worldwide [4]. However, critical-sized bone defects cannot be completely healed by the self-healing ability of the human body [5,6]. Hence, more than two million of bone graft implantations are performed annually around the world [7]. However, conventional bone grafts, including autografts and allografts, suffer from various drawbacks, such as limited availability and donor site morbidity [8,9,10]. To overcome these shortcomings, tissue-engineered bone substitutes prepared using a combination of novel biomaterial and fabrication techniques have emerged as a promising therapeutic alternative [6]. Among the various techniques used to fabricate tissue-engineered bone constructs, three-dimensional (3D) bioprinting has been widely explored as an effective alternative, which provides cell-friendly microenvironments with a designed shape and porosity that promote bone repair and regeneration [11,12]. The flexibility associated with the fabrication of tissue-engineered scaffolds customized via computer-aided design and medical imaging can be advantageous for personalized bone defect treatments [13]. However, 3D-bioprinted bone substitutes are still affected by significant issues, such as the limited availability of printable biomaterials that meet key requirements, including excellent printability, high mechanical integrity, low toxicity, and appropriate cell interactions [14]. Therefore, these limitations present a significant challenge to the robust and accurate fabrication of bioprinted bone scaffolds capable of promoting effective regeneration of the target tissues [15,16]. For instance, the thermoplastic behavior and relatively low melting point of polycaprolactone (PCL), an FDA-approved biodegradable synthetic polymer, enables its easy processing into bone scaffolds fabricated with extrusion-based 3D bioprinting techniques. However, despite the biocompatibility and ease of fabrication of PCL, its intrinsic hydrophobicity and lack of osteoinductivity inhibit cell attachment and osteogenic differentiation, frequently preventing successful therapy [17,18].

To overcome the limitations of homogeneous material-based scaffolds in bone tissue engineering, several studies have investigated the use of bioceramic/polymer composites to improve the mechanical and biological properties of bone grafts [19,20]. The primary advantage of these materials is their biomimetic composition, which efficiently mimics the natural inorganic and organic constituents of bone tissue [21]. Bioceramics, such as hydroxyapatite (HA), tricalcium phosphate (TCP), and bioactive glasses, have been commonly used in bioceramic/polymer composites due to their similarity with the mineral phase of the natural bone matrix [22]. Among bioceramics for bone tissue-engineered scaffolds, HA is one of the most extensively studied materials due to its excellent physicochemical properties, such as osteoconductivity, bioactivity, and resorbability, which can compensate the limitations of polymeric matrices [16,21,23]. The addition of HA to a polymer improves not only the osteogenic properties of the fabricated composite scaffold but also its rheological properties, enhancing both mechanical strength and shape fidelity [19].

Both biocompatible synthetic polymers, such as polylactic acid (PLA), polylactic-co-glycolic acid (PLGA), and PCL, and natural hydrogels, such as collagen, alginate, and gelatin, have been widely used as the organic component of bioceramic/polymer composites for bone tissue engineering applications [21]. However, the inherent limitations of synthetic polymers, such as lack of bioactivity and relatively high viscosity, can negatively affect the desired microenvironment for effective cellular interactions and the bioceramic contents (≤50%) [24]. In contrast, natural hydrogels can induce bioactive responses through the biofunctional molecules absorbed on their surface, which can enhance cell attachment, proliferation, and differentiation [16]. Moreover, natural hydrogels are frequently used for 3D bioprinting due to their shear-thinning properties and excellent bioactivity, but their poor mechanical properties can significantly limit effective bone repair in preclinical and clinical models [25,26]. Hence, several studies have attempted to improve the crosslinking and geometrical features of hydrogels to enhance the efficacy of bone defect treatments using bioprinted hydrogels [27,28].

To overcome the above limitations, multiple combinations of bioceramic/hydrogel composites, including α-TCP/gelatin, HA/alginate, and bioactive glass/gelatin, have been explored for bone tissue regeneration [19,21]. Although many studies have been performed on bioceramic/hydrogel composites, the fabricated composite scaffolds are mechanically and compositionally not compatible with natural bone due to the intrinsic limitations of hydrogels and the low bioceramic contents. Furthermore, bioprinting of composites with high bioceramic contents is challenging because of their high rheological resistance; hence, these materials are seldom employed in bioprinting. Therefore, in this study, gelatin-based composites incorporating large weight fractions (≥60%) of nanosized HA were used to provide an adequate biomimetic environment for bone tissue regeneration, with compositional and mechanical similarity to the natural bone matrix. For the precise fabrication of HA/gelatin composite scaffolds with high printing resolution and shape fidelity, the rheological properties of the composite materials were systematically assessed to optimize printing parameters, including pressure, temperature, and nozzle velocity. Furthermore, the bioprinted scaffolds were physicochemically characterized using X-ray diffractometry (XRD), Fourier transform infrared (FTIR) spectroscopy, X-ray photoelectron spectroscopy (XPS), and scanning electron microscopy (SEM) to confirm the composition and microstructure of the bioceramic/hydrogel composites. Moreover, the mechanical strength of HA/gelatin composites was measured using a compressive test. Finally, adipose-derived mesenchymal stem cells (ADMSCs) were seeded on the HA/gelatin composite scaffolds to assess their biocompatibility, cell proliferation, and osteoconductivity.

## 2. Results and Discussion

### 2.1. Rheological Properties of HA/Gelatin Composites

**Figure 1:**
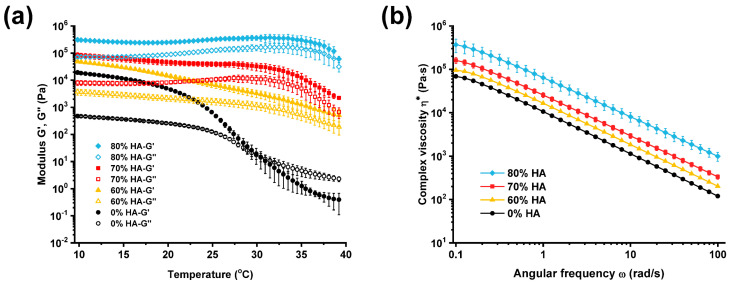
Rheological properties of HA/gelatin composites with different amounts of HA. (**a**) Shear storage and loss moduli as a function of temperature. (**b**) Complex viscosity as a function of angular frequency at 20 °C.

In 3D bioprinting applications, rheological properties are known to be significantly correlated with printing accuracy and shape fidelity [29,30,31]. Therefore, the rheological characteristics of HA/gelatin composites were investigated to predict the deposition quality and optimize the printing parameters before the additive fabrication of the composite bone scaffolds [32,33,34]. The influence of the temperature on the rheological properties of the HA/gelatin composites was assessed at four different concentrations of HA (0%, 60%, 70%, and 80%). Each group in Figure 1a exhibits a change in storage modulus (G’) and loss modulus (G”) as the temperature varied from 10 to 40 °C. While the G’ and G” curves of gelatin alone (without HA) crossed near 30 °C, indicating the sol–gel transition, the G’ values of HA/gelatin samples were higher than the G” values, and the corresponding curves in each group did not cross, reflecting the enhanced mechanical stability of the hydrogel upon the addition of the bioceramic. Overall, the G’ and G” values increased with increasing HA contents from 0 to 80%. The complex viscosities (η*) of the HA/gelatin composites were also measured at 20 °C with varying angular frequencies (ω), as shown in Figure 1b. The viscosity of the HA/gelatin composite increased with the amount of HA. In addition, all viscosity curves exhibited distinct shear-thinning behaviors without any plateau.

### 2.2. Fabrication of HA/Gelatin Composite Scaffolds

**Figure 2:**
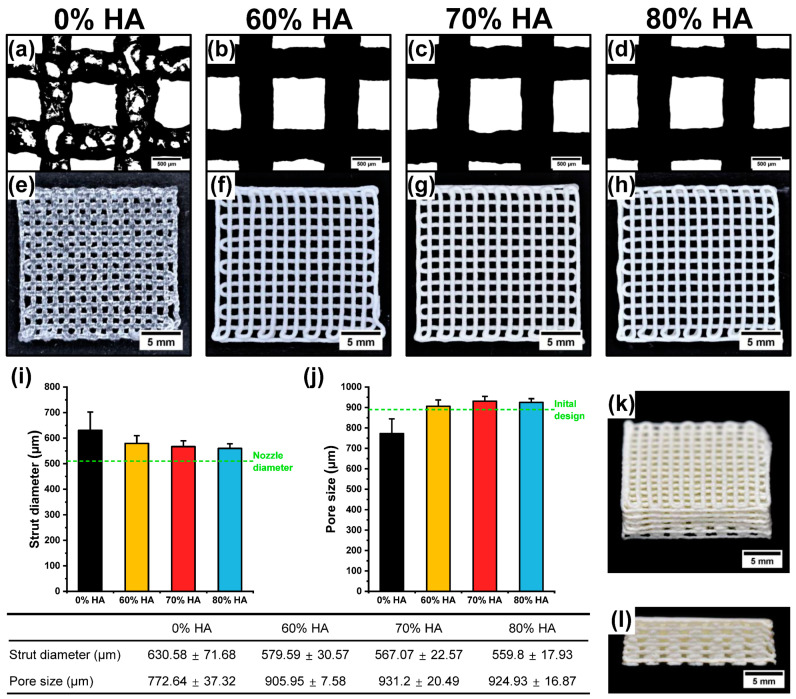
Printability assessment of HA/gelatin composites with different amounts of HA. Microscopic images (scale bar = 500 μm) and photographs (scale bar = 5 mm) of 3D-printed HA/gelatin composite scaffolds with (**a**,**e**) 0%, (**b**,**f**) 60%, (**c**,**g**) 70%, and (**d**,**h**) 80% HA. Statistical analysis of (**i**) strut diameters and (**j**) pore sizes. Photographs of 60% HA/gelatin composite scaffold under optimal printing conditions: (**k**) top and (**l**) side view (scale bar = 5 mm).

To evaluate the printability of each HA/gelatin composite, three-layered HA/gelatin composite scaffolds were initially fabricated with HA contents of 0%, 60%, 70%, and 80%, based on the rheological properties of each group (Figure 2a–h). In the case of 0% HA, gelatin showed significantly poor printability at both lower and higher temperatures than the sol–gel transition temperature (near 30 °C) due to its low shear moduli and over-gelation [35]. However due to the increased shear moduli and viscosities upon addition of HA, the HA/gelatin composites could be additively fabricated with excellent printability using an extrusion-based bioprinting technique. The strut diameter and pore size of the printed scaffolds were then analyzed to quantify their printing accuracy. The calculated strut diameter and pore size of the scaffolds with 60%, 70%, and 80% HA were similar to each other and comparable to ideal printing conditions (strut diameter: 510 μm; pore size: 890 μm). However, the scaffold prepared without HA exhibited a significantly inconsistent strut diameter and pore size, which were substantially different from the nozzle diameter and pore size of the initially designed structure (Figure 2i,j). These experimental results indicate that the incorporation of bioceramics such as HA into hydrogels can enhance their extrudability and printing accuracy, which is essential for the additive fabrication of tissue-engineered constructs [36]. In addition, the enhanced rheological properties enabled the HA/gelatin composite to be deposited in 12 layers with high shape fidelity (Figure 2k,l).

### 2.3. Chemical and Structural Properties of HA/Gelatin Composite Scaffolds

**Figure 3:**
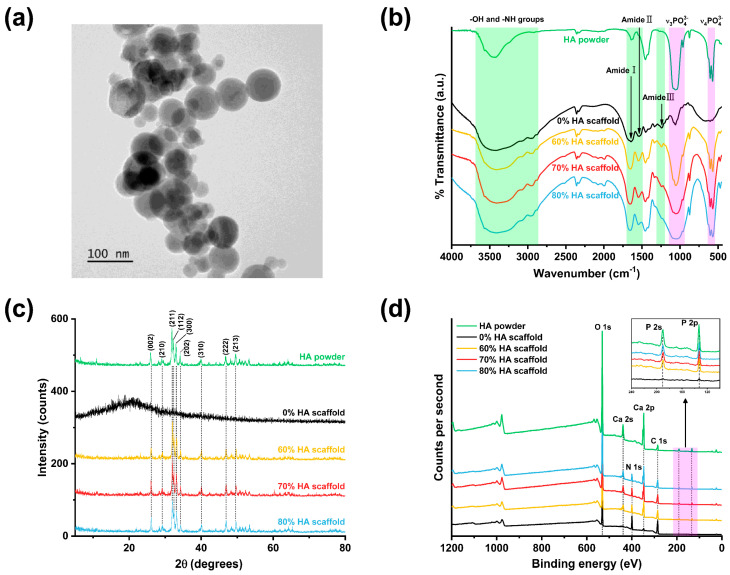
(**a**) TEM image of HA powder (scale bar = 100 nm). (**b**) FTIR, (**c**) XRD, and (**d**) wide-scan XPS spectra of HA/gelatin composites (0%, 60%, 70%, and 80% HA) and HA powder.

The morphology of HA powders was investigated by transmission electron microscopy (TEM), as shown in Figure 3a. The TEM images indicated the presence of spherical-shaped HA nanoparticles. The chemical structure of the HA/gelatin composite scaffolds was investigated using FTIR, XRD, and XPS. The FTIR spectra of all fabricated scaffolds display a series of amide and carboxyl bands corresponding to the chemical structure of gelatin (Figure 3b). The peaks at 1644, 1536, and 1239 cm^−1^ were attributed to the C=O stretching (amide I), N–H bending (amide II), and N–H stretching (amide III) vibrations, respectively [37], while the peak at 1450 cm^−1^ was associated with carboxyl groups [38]. For an effective analysis of the HA vibrational properties, the FTIR spectrum of pure HA powder was also measured and compared with those of the scaffolds. According to this analysis, the peaks at 603 and 575 cm^−1^ were attributed to the bending vibration of the phosphate group, while those at 1059, 1093, and 963 cm^−1^ were associated with the phosphate stretching vibration [39]. All fabricated scaffolds, except the HA-free gelatin scaffold, showed the characteristic peaks of the phosphate group, whose intensity increased slightly with the HA content.

The physical structures of the HA powder and fabricated HA/gelatin composite scaffolds were assessed using XRD (Figure 3c). Only the scaffold without HA showed a large amorphous hump around 2θ = 20°, which is the typical XRD pattern of pure gelatin, originating from its α-helix and triple-helical structure [40]. Distinct peaks were observed in the XRD patterns of the pure HA powder at 2θ values of 25.99° corresponding to the (002) reflection; 28.43° for reflections (102) and (210); 31.9° (triplet) for reflections (211), (112), and (300); and 34° for reflection (202). These are the principal diffraction peaks of HA, according to the JCPDS card no. 09-0432 [37]. Apart from the HA-free scaffold, all fabricated scaffolds containing HA exhibited distinct characteristic HA peaks.

The elemental compositions of the HA powder and fabricated HA/gelatin composite scaffolds were analyzed using XPS. As shown in Figure 3d, the spectra of all fabricated scaffolds incorporating HA showed the characteristic peaks of HA at 133, 199, 347, and 437 eV, attributed to P 2p, P 2s, Ca 2p, and Ca 2s states, respectively [41]. Furthermore, the intensity of these peaks increased with the HA content. The N 1s peak at 400 eV, indicating the presence of gelatin, was observed in the spectra of all scaffolds [42].

**Figure 4:**
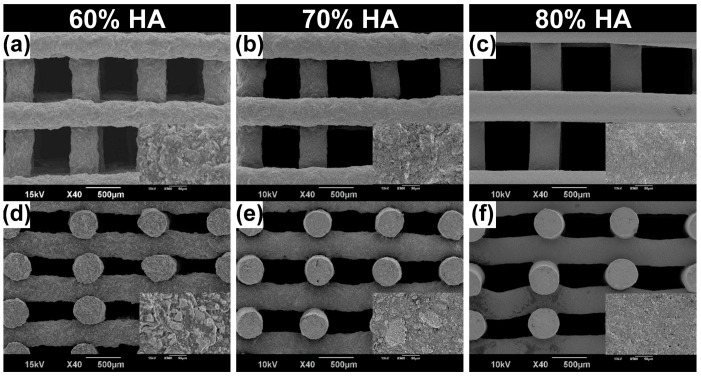
SEM images (scale bar = 500 μm) of HA/gelatin composite scaffolds with (**a**,**d**) 60%, (**b**,**e**) 70%, and (**c**,**f**) 80% HA contents. The insets show high-magnification (500×) images of the surface of the scaffold. (**a**–**c**) Top views; (**d**–**f**) side views.

The morphology and architecture of composite scaffolds containing HA were visualized using SEM (Figure 4). The scaffold pores were completely open and matched well with the predesigned structure of all three scaffolds. The composite scaffold with high HA content showed a relatively smooth surface compared to those with lower HA contents, at both low and high magnification. The roughness of the scaffold surface was strongly dependent on the HA and gelatin contents, which may be due to the influence of the degree of evaporation during the drying process [43].

### 2.4. Mechanical Properties of HA/Gelatin Composite Scaffolds

**Figure 5:**
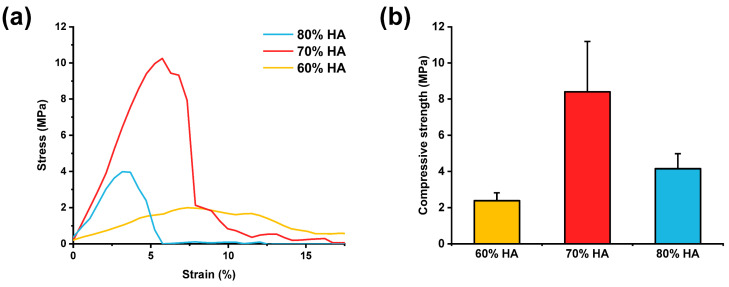
(**a**) Representative stress–strain curves and (**b**) compressive strengths of HA/gelatin composite scaffolds.

To quantitatively assess the role of HA on the mechanical properties of the HA/gelatin composite scaffold, compressive tests were performed with varying concentrations of HA, as shown in Figure 5a. The strain values at the maximum applied stress decreased with increasing contents of HA (approximately 7% for 60% HA, 5% for 70% HA, and 3% for 80% HA), indicating that the composite scaffolds became less ductile due to the interference of the bioceramic with the hydrogel crosslinking. The highest compressive strengths observed for 60%, 70%, and 80% HA contents were 2.3 ± 0.4, 8.4 ± 2.7, and 4.1 ± 0.8 MPa, respectively (Figure 5b). Moreover, the compressive strengths of all HA/gelatin scaffolds were comparable to that of cancellous bone [44,45]. The 70% HA scaffold, with similar composition to native bone, had a significantly higher compressive strength compared to the other samples. These results confirm the feasibility of using bioprinted bone scaffolds to mimic the mechanical properties and composition of native bone tissue, overcoming the limitations of current bioceramic/polymer composite scaffolds [46,47,48].

### 2.5. Biological Properties of HA/Gelatin Composite Scaffolds

#### 2.5.1. Analysis of Cell Proliferation and Attachment

**Figure 6:**
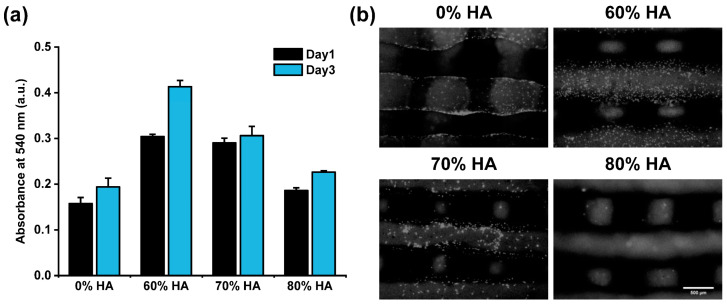
(**a**) Cell proliferation and (**b**) fluorescence microscopy images (scale bar = 500 μm) of Hoechst-stained ADMSCs attached on HA/gelatin composite scaffolds.

To assess the effect of the HA/gelatin composite scaffolds on cell proliferation and attachment, ADMSCs were cultured on the scaffolds. The cell proliferation in each scaffold was evaluated at days 1 and 3 using the 3-(4,5-dimethylthiazol-2-yl)-2,5-diphenyltetrazolium bromide (MTT) assay (Figure 6a). At both time points, all scaffolds containing HA showed higher absorbance values than those without HA. The highest cell proliferation was observed for the scaffold containing 60% HA, as indicated by a significantly higher absorbance value than that of the other three groups. These data imply that the presence of HA in the scaffolds can stimulate the proliferative response of ADMSCs.

To assess the cell attachment, we obtained fluorescence images of Hoechst-stained mesenchymal stem cells attached on the scaffolds (Figure 6b). Similar to the MTT results, the highest number of proliferated cells was detected on the surface of the scaffold with 60% HA. However, a relatively low number of cells were observed in the 80% HA scaffold compared to the others. The reduced cell attachment to this scaffold might be due to its relatively smooth surface (as shown in Figure 4) and reduced accessibility of cell-binding motifs owing to the relatively low gelatin proportion [49].

Many studies have demonstrated that the surface properties of a scaffold can affect cell attachment, proliferation, differentiation, and even morphology [50,51,52]. It is well known that the gelatin used in scaffolds can facilitate the exposure of cell-binding motifs (i.e., RGD) that enable biological interactions between cells and scaffolds [19]. Moreover, the introduction of hydroxyapatite alters the microscale surface roughness of the scaffold, which, in turn, can affect its cellular response, enhancing cell attachment and proliferation [51]. Therefore, the appropriate proportions of HA and gelatin must be adopted in the composite scaffolds to achieve effective bone repair.

#### 2.5.2. Osteogenic Differentiation of ADMSCs

**Figure 7:**
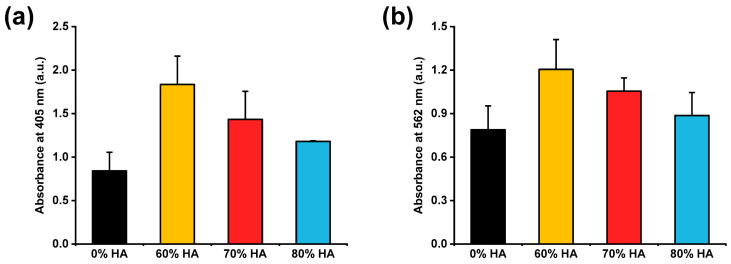
(**a**) ALP activity and (**b**) ARS absorbance of ADMSCs incubated on HA/gelatin composite scaffolds.

The osteogenic differentiation of ADMSCs was assessed by evaluating the alkaline phosphatase (ALP) activity and calcium deposition by alizarin red S (ARS) staining. The HA-containing scaffolds had a higher ALP activity than those without HA at day 14 (Figure 7a). Furthermore, the scaffolds with 60–80% HA had higher ARS absorbance values than the 0% HA scaffold at day 21 (Figure 7b). These results imply that the HA particles incorporated in the scaffolds can promote the osteogenic differentiation of ADMSCs, likely by enhancing the ALP activity of mesenchymal stem cells and stimulating the endogenous expression of osteogenic growth factors, such as bone morphogenetic proteins [16].

## 3. Conclusions

In this study, biomimetic composite bone scaffolds were successfully fabricated using extrusion-based bioprinting of gelatin composites containing large weight fractions of HA (60, 70, and 80%) with the aim to provide an adequate biomimetic environment for bone tissue regeneration, with compositional and mechanical properties similar to those of the natural bone matrix. The overall features of the bioprinted HA/gelatin composite scaffolds, including rheological, morphological, physicochemical, mechanical, and biological properties, were quantitatively assessed to identify the optimal conditions for both fabrication and therapeutic efficiency. The present results show that the bioprinted bioceramic/hydrogel scaffolds possess excellent shape fidelity; mechanical strength comparable to that of native bone; and enhanced bioactivity in terms of cell proliferation, attachment, and osteogenic differentiation. These experimental findings indicate a suitable alternative direction for the bioprinting-based fabrication of bioceramic/hydrogel-based scaffolds for bone repair.

## 4. Materials and Methods

### 4.1. Preparation of HA/Gelatin Composites

Nanosized hydroxyapatite powder (<200 nm particle size, nGimat) and type-A gelatin (porcine skin, Sigma Aldrich) were used to fabricate HA/gelatin composites. Gelatin powder was dissolved in distilled water in a rotational shaker at 50 °C and mixed with 4.5, 7, or 12 g of HA powder (Table 1). The mixture was placed in a planetary centrifugal mixer (AR-100, Thinky) for 1–2 min to produce homogeneous HA/gelatin composites.

### 4.2. Rheological Characterization

Rheological measurements of HA/gelatin composites were performed with a HR-2 (TA instruments) rheometer operating in oscillatory mode, using a 20 mm parallel plate with a 500 µm gap. An amplitude sweep test at 1 Hz was performed to determine the linear viscoelastic region before other rheological measurements. Temperature sweep tests were performed with a −5 °C/min temperature ramp from 40 to 10 °C at a 1 Hz frequency and 1% strain. In the frequency sweep tests, the angular frequency was increased from 0.1 to 100 rad/s at 20 °C with a 1% strain.

### 4.3. Fabrication of Composite Scaffolds

The HA/gelatin composite scaffolds were fabricated with a self-developed 3D bioprinting system. The nozzle temperature was set between 30 and 50 °C, according to the contents of each HA/gelatin composite. The printing velocity and extrusion pressure ranges were 5–15 mm/s and 200–400 kPa, respectively. The porous structure was generated in a 0°/90° orientation with a nozzle diameter of 0.51 mm. The distance between printed fibers was set to 1 mm for biological characterizations and 1.4 mm for the other measurements. The external dimensions of the scaffolds were set to 19.6 × 19.6 mm. The strut diameter and pore size of the printed scaffolds were quantified by optical image processing using the MATLAB software.

After fabrication, the scaffolds were immersed in a 0.25% *w*/*v* solution of glutaraldehyde for 10 min at room temperature to chemically crosslink the gelatin polymeric chains. To remove the remaining glutaraldehyde, the scaffolds were washed three times with distilled water.

### 4.4. Physicochemical and Structural Characterization

The chemical structure and composition of the HA powder and fabricated HA/gelatin composite scaffolds were analyzed by XRD (X’Pert-MPD, Philips, Eindhoven, The Netherlands), FTIR spectroscopy (FT-4100, Jasco, Tokyo, Japan), and XPS (AXIS Supra, Kratos, UK). The morphologies of the HA powder and HA/gelatin composite scaffolds were inspected by field-emission TEM (JEM-F200, JEOL, Tokyo, Japan) and low-vacuum SEM (JSM-6490LV, JEOL, Tokyo, Japan).

### 4.5. Mechanical Characterization

To assess their mechanical properties, the composite scaffolds were cut into small pieces (5.6 × 5.6 × 6 mm; 12 layers) and dried at 37 °C overnight. A compressive test was then conducted with a universal testing machine (LR5K Plus, Lloyd Instruments, Bognor Regis, UK) at a constant cross-head speed of 1 mm/min. Five samples of each scaffold were tested to ensure the reliability of the results.

### 4.6. Biological Characterization

#### 4.6.1. In Vitro Cell Culture

Rat adipose-derived mesenchymal stem cells (RASMD-01001, Cyagen, Santa Clara, USA) were used to evaluate the cellular behavior on the scaffolds. The fabricated scaffolds were prepared with six layers and cut to 5.6 × 5.6 × 3 mm pieces, then sterilized with ethanol and ultraviolet (UV) light. The cells (3 × 10^4^ cells/scaffold) were seeded on each scaffold and cultivated in Dulbecco’s modified Eagle’s medium (DMEM) supplemented with 10% fetal bovine serum (FBS), 1% penicillin–streptomycin (pen–strep), and 1% non-essential amino acids. The seeded scaffolds were incubated at 37 °C with 5% CO_2_. Before seeding the cells, the scaffolds were preincubated in a culture medium for 24 h. The cells from the fifth passage were used in all experiments.

#### 4.6.2. Cell Viability and Attachment

After 1 and 3 days of culturing on scaffolds, cell viability was evaluated using the MTT assay. The cell-seeded scaffolds were treated with 0.5 mg/mL MTT solution for 4 h at 37 °C. The scaffolds were then placed in dimethylsulfoxide (DMSO) for 30 min at room temperature to dissolve the MTT formazan. The solubilized formazan was measured in a microplate reader (Epoch, BioTek, Winooski, VT, USA) at 540 nm. Each time point was tested in triplicate. After 4 days of cell culture, cell attachment on the scaffold was evaluated by Hoechst 33,342 staining and fluorescent microscopy imaging (Eclipse Ts2, Nikon, Tokyo, Japan).

#### 4.6.3. Osteogenic Differentiation

ALP assay and ARS tests were performed to analyze the osteogenic differentiation of ADMSCs on the scaffolds. The cells were seeded as described above, incubated for 24 h, and transferred to osteogenic differentiation media consisting of DMEM supplemented with 10% FBS, 1% pen–strep, 50 μg/mL l-ascorbic acid, 10 mM β-glycerophosphate, 10 nM calcitriol, and 100 nM dexamethasone. After 14 days, the osteogenic activity was assessed by ALP assay. The scaffolds were washed with PBS and gently submerged in 1-step p-nitrophenyl phosphate (pNPP) solution; then, the absorbance was measured at a wavelength of 405 nm using a microplate reader.

Calcium mineralization was evaluated by ARS staining. After incubating them for 21 days, the scaffolds were washed with PBS and fixed with 70% cold ethanol at room temperature for 1 h. Then, the ethanol-fixed scaffolds were stained with ARS (pH 4.2) for 20 min. To quantify calcium mineralization, the scaffolds were incubated in 10 mM sodium phosphate buffer (pH 7.0) containing 10% cetylpyridinium chloride for 15 min, and the absorbance was measured at a wavelength of 562 nm. Three samples were tested for each incubation time.

## Figures and Tables

**Table 1:** HA content of HA/gelatin composites prepared in this study.

HA Content (wt%)	HA (g)	Gelatin (g)	Distilled Water (mL)
0	0	3	15
60	4.5	3	15
70	7	3	15
80	12	3	15

## Data Availability

The data that support the findings of this study are available upon reasonable request from the authors.
